# Impaired adaptation of energy intake induces severe obesity in aged mice on a high‐fat diet

**DOI:** 10.14814/phy2.13989

**Published:** 2019-02-01

**Authors:** Tadashi Okada, Yuichiro Mita, Hideyuki Sakoda, Masamitsu Nakazato

**Affiliations:** ^1^ Division of Neurology, Respirology, Endocrinology and Metabolism Department of Internal Medicine Faculty of Medicine University of Miyazaki Miyazaki Japan; ^2^ CREST (Japan) Agency for Medical Research and Development (A‐MED) Chiyoda‐ku Tokyo Japan

**Keywords:** Aging, energy intake regulation, HFD, inflammation

## Abstract

High‐fat diet (HFD) feeding induces inflammation in various tissues, including the nodose ganglion and hypothalamus, resulting in obesity and metabolic disorders. In this study, we investigated the effect of short‐term HFD on aged and young mice. Aged mice easily gained weight during short‐term HFD feeding, and required many days to adapt their energy intake. One‐day HFD in aged mice induced inflammation in the distal colon, but not in the nodose ganglion or hypothalamus. The anorexic effect of glucagon‐like peptide‐1 (GLP‐1) was attenuated in aged mice. Intraperitoneal administration of GLP‐1 did not induce expression of genes that regulate feeding in the hypothalamus of aged mice. mRNA expression of the gene encoding the GLP‐1 receptor (*Glp1r*) in the nodose ganglion was significantly lower in aged mice than in young mice. Our findings suggest that adaptation of energy intake regulation was attenuated in aged mice, causing them to become obese in response to short‐term HFD feeding.

## Introduction

The hypothalamus plays a key role on the regulation of food intake (Schwartz et al. 2000) and appetite (Suzuki et al. 2010). Many gastrointestinal peptide hormones are connected to various food‐related signals (Read et al. 1994) and transmit information to the central nervous system via the vagal afferent nerve. High‐fat diet (HFD) feeding induces inflammation in tissues including the nodose ganglion and hypothalamus (Naznin et al. 2015). One‐day HFD induces hypothalamic inflammation, and this effect is attenuated by celiac vagotomy, indicating that gut‐derived inflammatory signals are transmitted to the hypothalamus via the vagal afferent nerve (Waise et al. 2015). Thaler et al. showed that HFD induces hypothalamic cytokine gene expression after 3 days of HFD exposure, followed by a decline to baseline values from days 7 to 14, and a subsequent return to elevated levels by day 28 (Thaler et al. 2012). In a previous study, we showed that HFD induced inflammation in the hypothalamus and the nodose ganglion 1 day after the start of HFD feeding (Waise et al. 2015), but not after 2 weeks of HFD feeding (Naznin et al. 2018).

Aging alters the release of some peptide hormones. For example, plasma concentration of ghrelin, gastric‐derived orexigenic peptide, is reduced in older people (Rigamonti et al. 2002) and the basal and stimulated levels of the satiating hormone cholecystokinin are elevated (MacIntosh et al. 1999). On the other hand, plasma concentrations of glucagon‐like peptide‐1 (GLP‐1), one of the proglucagon peptide, and anorexigenic factor peptide YY do not differ as a function of age. In older men, the decline in testosterone induces an increase in the production of leptin which is the energy expenditure hormone (Wilson and Morley 2003), which in turn regulates appetite‐stimulating factor neuropeptide Y (NPY) and nitric oxide in the hypothalamus, resulting in a reduction of food intake (Morley et al. 1999).

Dipeptidyl peptidase 4 (DPP4) is an intrinsic membrane glycoprotein that cleaves N‐terminal dipeptides from a variety of substrates such as neuropeptides (Yazbeck et al. 2009). GLP‐1 and gastric inhibitory polypeptide (GIP) are released from the intestinal mucosa and responsible for postprandial insulin secretion (Drucker and Nauck 2006). DPP4 plays a major role in glucose metabolism by degraded of GLP‐1 and GIP.

In this study, we assessed the effect of short‐term HFD feeding in aged mice. We found that aged mice readily became obese on short‐term HFD, and required many days to adapt their energy intake. We analyzed the inflammation induced by one‐day HFD feeding in aged mice and compared it with the same experiments with young mice (Waise et al. 2015). One‐day HFD induced inflammation in the distal colon, but not in the nodose ganglion or hypothalamus. The anorexic effect by GLP‐1 was lower in aged mice than in young mice. GLP‐1 administration did not induce expression of genes that regulate feeding in the hypothalamus of aged mice. Our results indicated that aged mice are impaired in their ability to adapt to alteration of the nutrient composition of the diet, and consequently are likely to become obese in response to short‐term HFD.

## Materials and Methods

### Animals

C57BL/6J male mice were maintained in individual cages under controlled temperature (21–23°C) and light (light on: 08:00–20:00) conditions. Animals were maintained on a chow diet (CD) (12.3% fat, 59.2% carbohydrate, 28.5% protein, 3.44 kcal/g; CLEA Rodent Diet CE‐2; CLEA Japan). All animal experiments were approved by the Animal Care and Use Committee of the University of Miyazaki.

### Animal experiments

#### Comparison of HFD feeding in young and aged mice

Young (6 weeks old) and aged (14–18 months old) mice were maintained on CD or HFD (60% fat, 20% carbohydrate, 20% protein, 5.24 kcal/g; D12492; Research Diets) for 2 weeks. Body weight and 24‐h food intake were examined on days 1, 2, 3, 5, 7, 10, and 14.

#### One‐day HFD feedings at aged mice

Aged mice (*n* = 8) were used in experiments. Mice were fed CD or HFD ad libitum for a 24‐h period. Body weight, food intake for 24 h, blood glucose, and plasma insulin levels were measured.

### Measurements of blood glucose and plasma insulin levels

Blood was collected by tail‐prick, and blood glucose was measured using a glucometer (Terumo). Plasma insulin level was measured using the Morinaga Ultra‐Sensitive Mouse Insulin ELISA Kit (Morinaga Institute of Biological Science) as described previously (Naznin et al. 2017).

### Immunohistochemistry

Whole brain, nodose ganglion, and distal colon were immersed in 4% paraformaldehyde/phosphate‐buffered saline (PBS) for 24 h at 4°C, and then incubated for 24 h in phosphate buffer containing 20% sucrose, quickly frozen on dry ice, and cut into 8‐*μ*m slices with a cryostat (Leica CM3050S; Leica) at −20°C. Sections were washed in PBS, blocked in Serum‐Free Protein Block (DAKO) for 10 min at room temperature, and then incubated overnight at 4°C with rabbit anti–ionized calcium binding adaptor molecule 1 (Iba1) (1:1000; WAKO), goat anti–Toll‐like receptor 4 (TLR4) (1:100; Santa Cruz Biotechnology), or rabbit anti‐mucin 2 (MUC2) (1:50; Santa Cruz Biotechnology). Sections were washed in PBS, and then incubated with Alexa Fluor 488–conjugated anti‐rabbit (1:1000; Invitrogen) or Alexa Fluor 594–conjugated anti‐goat (1:1000; Invitrogen) antibody for 1 h at room temperature. Nuclei were stained with DAPI. Images were captured on an Olympus AX‐7 fluorescence microscope or a Confocal Microscope C2 (Nikon). TLR4 and MUC2 pixel intensities were determined automatically using the cellSens Dimension imaging software (Olympus). Iba1‐positive cells were counted manually using the same software. Quantitation was performed in a blinded fashion.

### Real‐time polymerase chain reaction (RT‐PCR)

Hypothalamus, nodose ganglion, liver, epididymal fat, and mesenteric fat were taken from anesthetized CD‐ or HFD‐fed mice. Distal colons were washed in PBS, and the epithelial cells of the luminal side were collected. Total RNA was extracted with the RiboPure™ kit (Ambion). RT‐PCR was performed on a Thermal Cycler Dice Real Time System II (Takara Bio Inc.) with SYBR Premix Ex Taq (2×) (Takara Bio) and the following primers are shown in Table 1. Each mRNA level was normalized against the level of *Gapdh* mRNA in the same sample.

**Table 1:** Primer sets for RT‐PCR

gene	Forward	Reverse
*Emr1*	5′‐GAGATTGTGGAAGCATCCGAGAC‐3′	5′‐GACTGTACCCACATGGCTGATGA‐3′
*Iba1*	5′‐AGCTGCCTGTCTTAACCTGCATC‐3′	5′‐TTCTGGGACCGTTCTCACACTTC‐3
*Il6*	5′‐CCACTTCACAAGTCGGAGGCTTA‐3′	5′‐CCAGTTTGGTAGCATCCATCATTTC‐3′
*Tnf*	5′‐TATGGCCCAGACCCTCACA‐3′	5′‐GGAGTAGACAAGGTACAACCCATC‐3′
*Tlr4*	5′‐GGAAGTTCACATAGCTGAATGAC‐3′	5′‐CAAGGCATGTCCAGAAATGAGA‐3′
*Itgam*	5′‐CCACTCATTGTGGGCAGCTC‐3′	5′‐GGGCAGCTTCATTCATCATGTC‐3′
*Itgax*	5′‐AGGTCTGCTGCTGCTGGCTA‐3′	5′‐GGTCCCGTCTGAGACAAACTG‐3′
*F4/80*	5′‐CTCTGTGGTCCCACCTTCAT‐3′	5′‐GATGGCCAAGGATCTGAAAA‐3′
*Mpo*	5′‐CTGCCTCATTGGCACTCAGTTTA‐3′	5′‐GGTGATGCCAGTGTTGTCACAG‐3′
*Npy*	5′‐CAGATACTACTCCGCTCTGCGACACTACA‐3′	5′‐TTCCTTCATTAAGAGGTCTGAAATCAGTGTC‐3′
*Agrp*	5′‐TTTGTCCTCTGAAGCTGTATGC‐3′	5′‐GCATGAGGTGCCTCCCTA‐3′
*Crh*	5′‐GAATTTCTTGCAGCCGGAGC‐3′	5′‐CAGCGGGACTTCTGTTGAGA‐3′
*Pomc*	5′‐ACCTCACCACGGAGAGCA‐3′	5′‐GCGAGAGGTCGAGTTTGC‐3′
*Ghsr*	5′‐AATGCCCTGGTCACCCAGA‐3′	5′‐TGAGGACCATAACCTTGACTTTGAG‐3′
*Glp‐1r*	5′‐ATGGTGGCTATCCTGTACTGCTTTG‐3′	5′‐GCTGCTGGTGGGACACTTGA‐3′
*Gapdh*	5′‐TCAAGAAGGTGGTGAAGCAG‐3′	5′‐TGGGAGTTGCTGTTGAAGTC‐3′

### Food intake experiments

Young and aged mice were maintained on CD ad libitum. Before experiments, young and aged mice were acclimatized by intraperitoneal injection of saline once per day for 5 days. Subsequently, the mice received intraperitoneal injection of GLP‐1 (75 nmol/kg in 50 μL saline, PEPTIDE INSTITUTE, INC) or saline (50 μL) after fasting for 16 h (18:30–10:30), and then food intake of CD was measured for 1 h (10:30–11:30).

### Analysis of genes that regulate feeding

Young and aged mice were maintained on CD ad libitum. After 1 day of HFD, the hypothalamus was collected from young (*n* = 5) and aged (*n* = 5) mice. To examine the effect of GLP‐1 on the expression of genes that regulate feeding, young (*n* = 5) and aged (*n* = 5) mice were fasted for 16 h, injected intraperitoneally with GLP‐1 or saline, and the nodose ganglion and hypothalamus were collected 2 h later. Total RNA extraction and RT‐PCR were performed as described in section 2.5.

### Statistical analysis

Data are expressed as means ± S.E.M. Statistical analyses were performed using the Excel and IBM SPSS software. Differences between two groups were assessed by two‐tailed unpaired Student's t‐test. Differences among more than two groups were assessed by analysis of variance (ANOVA) as described in the figure legends. *P* < 0.05 was considered to indicate statistical significance.

## Results

### HFD feeding phenotypes in young and aged mouse

**Figure 1:**
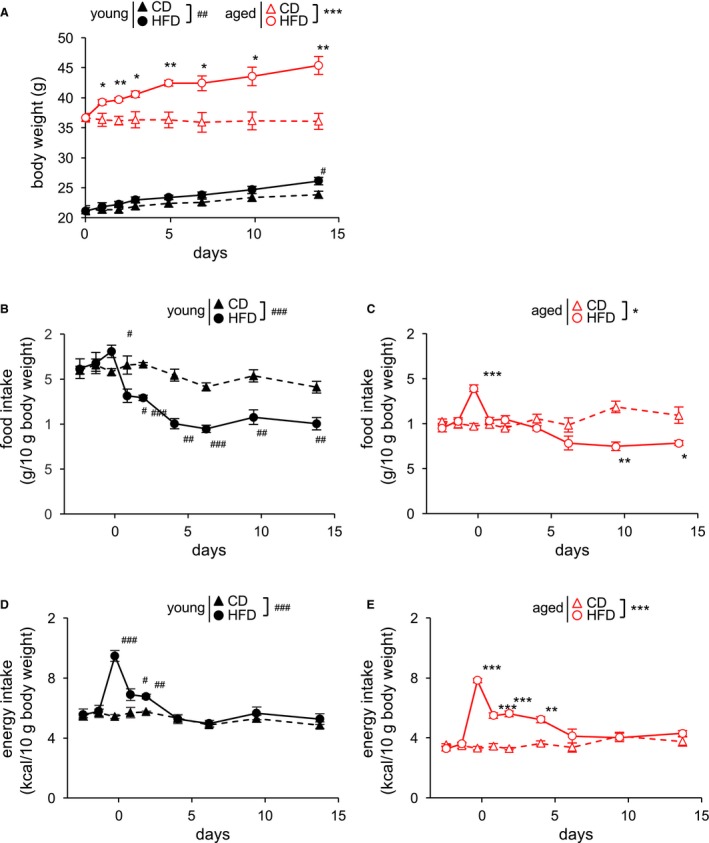
Time course of body weight change in CD‐ or HFD‐fed young and aged mice (A). Time course of 24‐h food intake in CD‐ or HFD‐fed young (B) and aged mice (C). Time course of energy intake in CD‐ or HFD‐fed young (D) and aged mice (E). “Day 0” indicates the day the diet was switched. Data for each time point are expressed as means ± SEM. *P <* *0.05, **P <* *0.01, ***P <* *0.001 versus CD‐fed aged mice; ^#^ *P *<* *0.05, ^##^ *P *<* *0.01, ^###^ *P *<* *0.001 versus CD‐fed young mice. Differences between two groups were assessed by two‐tailed unpaired Student's *t*‐test. *P <* *0.05, ***P <* *0.001 vs CD‐fed aged mice; ^##^ *P *<* *0.01, ^###^ *P *<* *0.001 versus CD‐fed young mice (ANOVA with Tukey's post hoc test).

We assessed the short‐term HFD feeding effect in both young and aged mice. In young mice, body weights were significantly higher in HFD‐fed mice than in CD‐fed mice after 2 weeks of HFD feeding (Fig. 1A). In aged mice, one‐day HFD increased body weight significantly in comparison with CD‐fed mice (Fig. 1A). Relative body weights (HFD/CD) were 1.096 ± 0.026 (young) and 1.259 ± 0.041 (aged) after 2 weeks of HFD feeding. In both young and aged mice, food intake was significantly higher in HFD‐fed mice 1 day after the initiation of HFD feeding (Fig. 1B and C). However, in young mice, food intake drastically decreased, and the amount of food consumed was significantly lower than on CD from day 2 to day 14 (Fig. 1B). On the other hand, in aged mice, food intake decreased more slowly (Fig. 1C). In young mice, energy intake was significantly higher on HFD than on CD from day 1 to day 3 (Fig. 1D), whereas in aged mice, energy intake was significantly higher from day 1 to day 5 and tended to be higher on day 7 (Fig. 1E). The total amount of energy intake over 2 weeks was significantly larger in HFD‐fed mice than in CD‐fed mice (young: CD: 167.73 ± 6.69 kcal, HFD: 188.68 ± 3.61 kcal; aged: CD: 181.05 ± 7.78 kcal, HFD: 270.59 ± 9.99 kcal).

### Characteristics and immunohistochemical analysis of one‐day HFD in aged mice

Characteristics and blood parameters of CD‐ or HFD‐fed aged mice are shown in Table 2. One‐day HFD significantly increased body weight, body weight gain, food intake, energy intake, and plasma insulin.

**Table 2:** Characteristics and parameters of CD‐ or HFD‐fed aged‐mice

	CD	HFD
Initial body weight (g)	35.55 ± 0.23	35.96 ± 0.29
Final body weight (g)	35.53 ± 0.19	38.40 ± 0.37^a^
Body weight gain (g)	−0.01 ± 0.26	2.44 ± 0.16^a^
24‐h food intake (g)	3.70 ± 0.20	5.71 ± 0.23^a^
24‐h energy intake (kcal)	12.74 ± 0.64	29.91 ± 1.18^a^
Blood glucose (mg/dl)	147.33 ± 4.13	144.50 ± 6.40
Plasma insulin (ng/ml)	2.61 ± 0.47	8.94 ± 1.05^a^

Data are shown as means ± SEM (*n* = 8).

a *P* < 0.001 versus CD.

In a previous study, we reported that the effects of one‐day HFD feeding in young mice (Waise et al. 2015). We showed one‐day HFD feeding induced TLR4 and MUC2 expression in the distal colon and increased Iba1‐positive cells, indicating activated macrophage/microglia, in the nodose ganglion and hypothalamus. Accordingly, we investigated the one‐day HFD feeding in aged mice and compared with the previous report (Waise et al. 2015).

**Figure 2:**
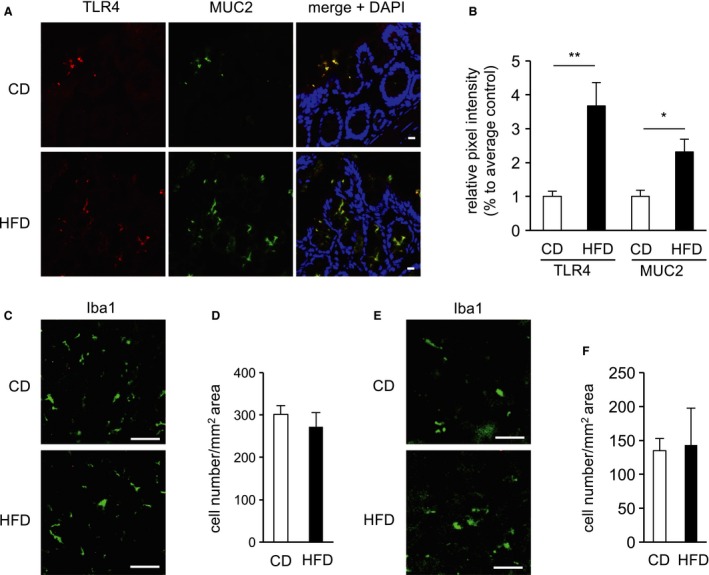
Analysis of inflammatory markers. (A) Immunohistochemical analysis of TLR4 (red), MUC2 (green) and Nuclei (blue) in the distal colon of one‐day CD‐ or HFD‐fed aged mice. Scale bars, 50 *μ*m. (B) Relative expression of TLR4 and MUC2 in the distal colon. The hypothalamus and nodose ganglion were immunostained with anti‐Iba1 antibody. Analysis of activated microglia in the hypothalamus (C, D) and macrophages in the nodose ganglion (E, F). Quantitation of Iba1‐positive cells in the hypothalamus (D) and in the nodose ganglion (F). Scale bars, 50 *μ*m. Values are means ± SEM. *P < 0.05, **P < 0.01 versus CD‐fed mice.

Both TLR4 and MUC2 levels in the distal colon were significantly higher in HFD‐fed than in CD‐fed aged mice (Fig. 2A and B). The number of Iba1‐positive cells did not differ between CD‐ and HFD‐fed mice in either the hypothalamus or the nodose ganglion (Fig. 2C, D, E, and F).

### Analysis of inflammatory markers by RT‐PCR

Previously, we reported that one‐day HFD feeding induces inflammation in some tissues of young mice (Waise et al. 2015). Under these conditions, the mRNA levels of inflammatory markers (*EGF‐like module‐containing mucin‐like hormone receptor‐like 1* (*Emr1*), *Iba1*,* interleukin‐6* (*Il6*), and *tumor necrosis factor‐α* (*Tnf*)) were significantly elevated in the nodose ganglion and hypothalamus. In addition, the mRNA levels of *Emr1*,* integrin subunit alpha X* (*Itgax*) (*CD11c*), and *Tnf* in the distal colon, *myeloperoxidase* (*Mpo*) in the liver and epididymal fat, and *integrin subunit alpha M* (*Itgam*) (*CD11b*), *Tnf*, and *Mpo* in mesenteric fat were also significantly elevated. Hence, we examined the effects of one‐day HFD on inflammation by evaluating the levels of these inflammatory markers in aged mice and compared them with the previous study (Waise et al. 2015).

**Figure 3:**
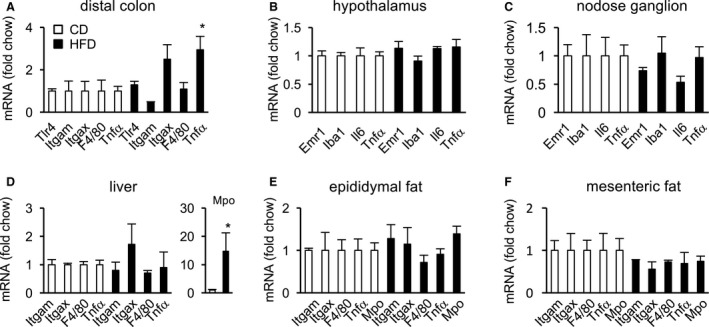
Effect of one‐day HFD on inflammatory mRNA expression in the distal colon (A), hypothalamus (B), nodose ganglion (C), liver (D), epididymal fat (E), and mesenteric fat (F) of CD‐ or HFD‐fed aged mice. mRNAs were normalized against *Gapdh* expression and are presented as fold change relative to CD. Values are means ± SEM. *P < 0.05 versus CD.

The mRNA level of *Tnf* in the distal colon was significantly higher in HFD‐fed mice than in CD‐fed mice (Fig. 3A). *Tlr4* and *Itgax* (*CD11c*) mRNA levels also tended to be higher in HFD‐fed mice. The mRNA levels of inflammatory markers (*Emr1*,* Iba1*,* Il6*, and *Tnf*) did not differ between CD‐ and HFD‐fed aged mice in either the hypothalamus or the nodose ganglion (Fig. 3B and C). The *Mpo* mRNA level in the liver was significantly higher in HFD‐fed aged mice than in CD‐fed aged mice (Fig. 3D), and tended to be higher in epididymal fat of HFD‐fed mice (Fig. 3E). The mRNA levels of *Itgam* (*CD11b*), *Itgax*,* F4/80*,* Tnf*, and *Mpo* did not differ between CD‐ and HFD‐fed aged mice in mesenteric fat (Fig. 3F).

### Analysis of expression of genes that regulate feeding after one‐day HFD or GLP‐1 injection

**Figure 4:**
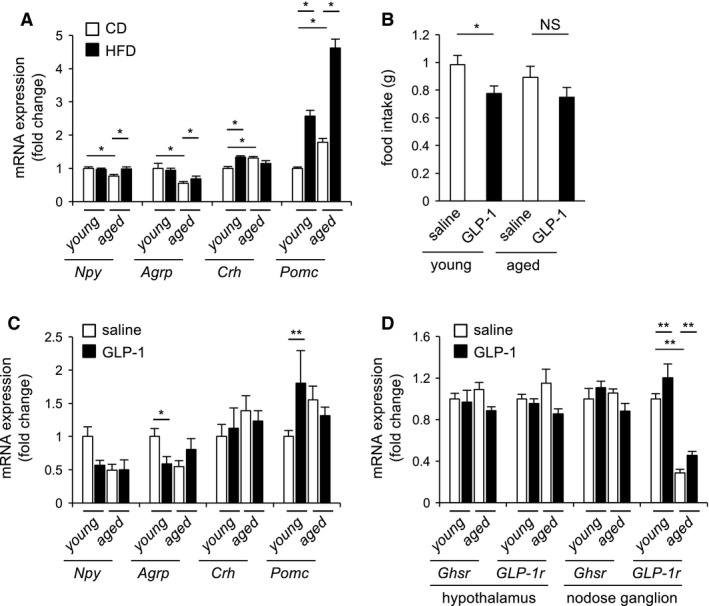
Analysis of GLP‐1 anorexic effect in young and aged mice. (A) mRNA levels of genes that regulate feeding in the hypothalamus of CD‐ or HFD‐fed young and aged mice. (B) One‐hour food intake after 16‐h fasting, measured with or without GLP‐1. (C) mRNA levels of genes that regulate feeding in the hypothalamus of young and aged mice, treated with or without GLP‐1. (D) mRNA levels of *Ghsr* and *Glp‐1r* in the nodose ganglion and hypothalamus of young and aged mice, treated with or without GLP‐1. Values are means ± SEM. *P < 0.05, **P < 0.01 (ANOVA with Tukey's post hoc test).

The mRNA levels of *Npy* and *Agouti‐related peptide* (*Agrp*) in the hypothalamus were significantly lower in aged mice than in young mice, whereas *corticotropin‐releasing hormone* (*Crh*) and *Pro‐opiomelanocortin* (*Pomc*) mRNA levels were significantly higher in the hypothalamus of aged mice (Fig. 4A). One‐day HFD induced mRNA expression of *Crh* and *Pomc* in the hypothalamus of young mice, and expression of *Npy*,* Agrp*, and *Pomc* in the hypothalamus of aged mice (Fig. 4A).

GLP‐1 administration significantly decreased food intake in young mice, but this anorexic effect was attenuated in aged mice (Fig. 4B).

Intraperitoneal administration of GLP‐1 down‐regulated *Agrp* mRNA expression, and increased *Pomc* mRNA expression in the hypothalamus of young mice (Fig. 4C). On the other hand, expression of genes that regulate feeding was not altered in the hypothalamus of aged mice following GLP‐1 administration (Fig. 4C). The *Glp1r* mRNA level in the hypothalamus did not differ between young and aged mice, but the level in the nodose ganglion was significantly lower in aged mice (Fig. 4D). The mRNA levels of *growth hormone secretagogue receptor* (*Ghsr*) and *Glp1r* did not change in the hypothalamus of either young or aged mice following GLP‐1 administration (Fig. 4D). By contrast, administration of GLP‐1 induced *Glp1r* expression in the nodose ganglion in both young and aged mice (Fig. 4D).

## Discussion

We assessed the effect of short‐term (one‐day and 2‐week) HFD feeding in young and aged mice. The results revealed that relative body weight increased 9.6% (young mice) and 25.9% (aged mice) over the 2‐week period. In chronically (5 months) HFD‐fed animals, body mass is significantly greater in young mice than in aged mice (Tucsek et al. 2014). Thus, aged mice become obese more easily than young mice in response to short‐term HFD feeding, whereas chronic HFD feeding causes greater body weight gain in young mice.

Thaler et al. (2012) showed that HFD induces a complex “on–off–on” pattern in cytokine gene expression in rat hypothalamus. In a previous study, we showed that one‐day HFD feeding induces TLR4 and MUC2 expression as a protective mechanism in the distal colon, as well as expression of inflammatory markers (*Emr1*,* Iba1*,* Il6*, and *Tnf*) in the nodose ganglion and the hypothalamus, in young mice (Waise et al. 2015). In this study, we found that one‐day HFD feeding of aged mice induced TLR4 and MUC2 expression in the distal colon. However, macrophage/microglia activation and expression of inflammatory markers (*Emr1*,* Iba1*,* Il6*, and *Tnf*) did not increase in the nodose ganglion or hypothalamus. Toll‐like receptors play roles in pathogen recognition and innate immune response by activating various inflammatory signaling pathways (Jialal et al. 2014). Fatty acids increase TLR4 expression and activity in a mouse model of diet‐induced obesity (Shi et al. 2006). Hypothalamic microglia, as a sensor of HFD, orchestrate inflammatory processes in the hypothalamus (Valdearcos et al. 2014). Our results reveal differences in the inflammatory response to HFD between young and aged mice. In aged mice, HFD induced an inflammatory response in the gut, but neuronal inflammation was not induced by one‐day HFD feeding. The aged innate immune system was activated and resulted in dysregulated inflammation and elevated basal inflammation in aged mice and humans (Shaw et al. 2013). It is possible that an elevation of basal inflammation in aged mice related the inflammatory response by HFD feeding.

Aging is associated with a decline in food intake (Morley 2001). In this study, the amount of food intake per body weight was smaller in CD‐fed aged mice than in CD‐fed young mice. Moreover, aged mice required a longer period of time than young mice to adapt to alteration of the nutrient composition of their chow. Appetite‐suppressing factors are upregulated, and appetite‐stimulating factors are attenuated, in aged rats (Akimoto and Miyasaka 2010). In this study, we found that expression of appetite‐stimulating factors *Npy* and *Agrp* were significantly lower in the hypothalamus of aged mice, whereas expression of appetite‐suppressing factors *Crh* and *Pomc* were significantly higher. In aged mice, the *Glp1r* mRNA level was significantly lower in the nodose ganglion, indicating attenuation of the anorexic effect of GLP‐1. Indeed, administration of GLP‐1 did not induce expression of genes that regulate feeding in aged mice. Overall, our results suggest that alteration in the expression of genes responsible for regulating feeding caused a decline in food intake and attenuation of energy intake adaptation in aged mice.

DPP4 degraded GLP‐1 and resulted regulates insulin secretion (Dominguez Avilla et al. 2017). Serum DPP4 concentration or DPP4 activity was negatively associated with age (Dimitrijevic et al. 2010; Lamers et al. 2011). These results suggest that GLP‐1 sensitivity is increased in aged mice. However, our result showed that anorexic effect by GLP‐1 administration was attenuated in aged mice. This result suggests that attenuation of anorexic effect by GLP‐1 administration in aged mice was not related to GLP‐1 degradation by DPP4.

In conclusion, our results suggested that gut‐derived signaling via the vagus nerve is attenuated in aged mice, obscuring subsequent regulation of food intake. Consequently, aged mice became obese more readily than young mice in response to short‐term HFD feeding. Based on our findings, we propose that control of appetite‐regulating gene expression in the nodose ganglion represents a promising therapeutic target in obese patients.

## Conflict of Interest

None declared.
